# Allen's Continuous Gum Work

**Published:** 1870-06

**Authors:** T. Hasbrouck


### ARTICLE VI.

### *Allen's Continuous Gum Work.*

By T. Hasbrouck, D. D. S.

There is so little said or written on this subject, that it seems to have been almost entirely neglected of late, not only in the dental schools, but in the columns of the journals. Continuous gum with a platina plate properly put up is, in the opinion of the writer, the most perfect thing in the way of artificial dentures that has ever been produced. This will, no doubt, appear to some as a very broad assertion, on account of their having seen very ugly looking specimens of the work in some mouths, and other objections have been its weight and liability to fracture when dropped. Others, that are not unfrequently urged are, that it is impossible to mend it after it is once broken, and that the plate will warp in baking, and consequently be a misfit when completed. All of these objections would be good ones if they had any foundation in fact, which any man who has a thorough knowledge of the work can easily demonstrate to be untrue.

We have seen a great number of cases of this work that were improperly made, and such coming under the observation of any good dentist, would very naturally give him the impression that, practically, continuous gum work is worthless, unless he happened to know how it *should* be done. There is but one way to do anything, and that is to do it right, and if this work is done in that way, it will be just as strong and durable as any, and much more natural and cleanly than any I have ever yet seen.

The plate, for a practical case, should be about No. 28, by gauge, and the French platina is preferable on account of its being smoother, brighter, and less likely to have cracks and fissures in it than that which is made from scraps and rolled out. The plate should be swaged in the same manner as an ordinary metal plate, being careful to keep the base metals from it in annealing. Get the articulation the same as in any case, and the teeth can be placed in most any position required. After they are arranged on the wax as desired, it is well to put plaster enough around the outside to hold them firmly in position while being backed and soldered.

Then invest in plaster and asbestos, first putting a platina wire across the heel of the plate to keep it from warping or springing while being baked. The backing should consist of about three separate pieces of platina, and cannot be too stiff or strong; solder with pure gold. It should not be soldered in the furnace, as the teeth will be very likely to be etched and spoiled by overheating while in the investment.

Heat them to a cherry-red in the furnace, and then melt the solder with the blow-pipe. After cooling off remove the investment, taking care to preserve the base with the wire in it to bake the piece on afterward. Put the piece in acid to remove the borax, and then wash thoroughly with soap. The case is then ready for the body. After the first coat, the cracks and fissures caused by the shrinkage must be carefully filled, and it will come out of the furnace the second time smooth and ready for the enamel, which can be put on thick or thin to suit the case, and shaded as desired.

The baking and furnace work is the most difficult part, and can only be learned by practice. There are many little annoyances that the beginner has to put up with, and gasing is perhaps the worst one. If it is heated up too fast it will snap and fly. If the case is gased, it is ruined, and might as well be made over at once. It will look blue, and be rough and spongy. A little practice and instruction from any one who understands it will enable one to overcome all these difficulties. As for the work breaking down easily, it will not do it in the mouth, and is no more likely to fracture than any other, unless dropped on marble or some other hard substance. If a patient has the misfortune to drop and break them, they can be repaired just as easily as any other work, and can be mended to look well, though, generally, after being mended two or three times they are not so strong as at first. The same may be said of almost any work.

To those who are fortunate enough to know how to do this work, this will, of course, be without interest. It is meant for those who do not, of whom, I am well aware, there are a great many in our profession. I do not wish to convey the idea that continuous gum is better than anything else, but have said these few words, hoping that there may be some interest awakened in its behalf, and am very sure that whoever takes it up, masters it, and uses it properly in his practice, will never have reason to regret it. I would not recommend its use for partial plates, but for entire upper, or under, or both, I think it is without any equal in most cases.—*The Dental Times.*

---

